# Antibacterial properties of an acrylic resin containing curcumin nanoparticles: An in vitro study

**DOI:** 10.34172/joddd.2022.032

**Published:** 2022-11-15

**Authors:** Pegah Khamooshi, Maryam Pourhajibagher, Ahmad Sodagar, Abbas Bahador, Badreddin Ahmadi, Sepideh Arab

**Affiliations:** ^1^Department of Orthodontics, Tehran University of Medical Sciences, Tehran, Iran; ^2^Medical Bacteriology and Dental Research Center, Tehran University of Medical Sciences, Tehran, Iran; ^3^Oral Microbiology Laboratory, Department of Microbiology, School of Medicine, Tehran University of Medical Sciences, Tehran, Iran; ^4^Faculty of Art and Architecture, Tarbiat Modares University, Tehran, Iran

**Keywords:** Acrylic resin, Antimicrobial, Curcumin, Nanoparticles, Poly (methyl methacrylate)

## Abstract

**Background.** Microbial accumulation is still a significant problem with removable acrylic appliances. This study aimed to assess the antimicrobial properties of a self-cured acrylic resin containing curcumin nanoparticles (CNPs).

**Methods.** This in vitro study used 48 acrylic discs containing 0.5%, 1%, and 2% CNPs. The antimicrobial properties of the discs against *Streptococcus mutans, Streptococcus sanguinis, Lactobacillus acidophilus,* and *Candida albicans* were evaluated using disc agar diffusion (DAD), eluted component, and biofilm inhibition tests. The growth inhibition zones were measured, and the colonies were counted after 1, 3, and 7 days.

**Results.** DAD test showed that none of the curcumin nanoparticle concentrations caused growth inhibition zones for any microorganisms. All the concentrations were effective against all four microorganisms in the biofilm inhibition test except 0.5% for *L. acidophilus*. In the eluted component test, solutions containing 2% concentration had maximum growth inhibition of all the groups at all time intervals. An increase in curcumin nanoparticle concentration from 0.5% to 1% was effective only against *C. albicans*.

**Conclusion.** Generally, CNPs in all concentrations were effective against the biofilms of all four microorganisms assessed in this study. Therefore, incorporating 2% CNPs into acrylic resin seems suitable for clinical use.

## Introduction

 Poly (methyl methacrylate) (PMMA) is commonly used in dentistry to fabricate full and partial dentures, temporary restorations, and removable orthodontic appliances such as functional appliances and retainers. Acrylic resins are mainly composed of PMMA, poly(ethyl methacrylate), and copolymers.^1,2^ PMMA is easy to use and economically reasonable. However, the most important problem with PMMA is its high potential for microbial plaque accumulation since food particles can lodge in the porosities of the acrylic surface. Microbial plaque accumulation can lead to decalcification and caries in tooth structure and/or gingival inflammation.^3-5^ Microbial plaque removal by mechanical and chemical procedures highly depends on patients’ cooperation and could be compromised in non-cooperative patients. Therefore, directly incorporating antimicrobial agents into acrylic could be a wise solution.^6-8^

*Streptococcus mutans*, *Streptococcus sanguinis*, and *Lactobacillus acidophilus* are mainly responsible for biofilm formation, caries development, and gingivitis. These bacteria are the main constituents of dental plaque as well. *Candida albicans* is the most common fungal species responsible for denture stomatitis.^9-11^ *S. mutans* is responsible for the initiation of caries, while *L. acidophilus* is responsible for the progression of carious lesions. *S. sanguinis* results in decreased *S. mutans* counts in the oral cavity since there is an equilibrium between these two microorganisms.^12,13^

 Several nanoparticles such as silver, silicon dioxide, titanium dioxide, zinc, and platinum have been successfully added to the acrylic resin to confer antimicrobial activity.^2,14-16^

 However, the abovementioned additives mainly include mineral ions, raising some biological concerns. Therefore, researchers are searching for organic products with optimal antimicrobial properties against cariogenic microorganisms.

 Curcumin is the effective substance of *Curcuma longa* from the *Zingiberaceae* family, which accounts for 2‒8% of the constituents of turmeric and is responsible for its golden yellow color and unique properties. It has insignificant toxicity and a wide range of anti-oxidative, anti-inflammatory, antimicrobial, and anti-cancer properties.^17-19^

 In recent years, nanotechnology has revolutionized the properties of materials. Nanoparticles have a high surface-to-volume ratio due to their small size. This property enhances their ability to form covalent bonds and creates specific characteristics.^20,21^

 Considering the above, this study aimed to assess the antimicrobial properties of self-cured acrylic resin containing different concentrations of CNPs against *S. mutans*, *L. acidophilus*, *S. sanguinis*, and *C. albicans.*

## Methods

 Forty-eight acrylic discs containing 0.5%, 1%, and 2% CNPs were fabricated and sterilized. A control group without CNPs was also considered. Thus, the antimicrobial efficacy of a control group without CNPs and three different concentrations of CNPs was evaluated against four types of microorganisms. With three repetitions, a total of 48 samples were used.

###  Preparation of CNPs

 Curcumin (Sigma Aldrich, USA) was dissolved in 1% acetic acid and water. The pH of the solution was adjusted to 4.6‒4.8 using NaOH. Afterward, 3 mL of this solution was added to 1 mL of tri-polyphosphate and stirred by a magnetic stirrer with high intensity. It was then centrifuged at 9000 rpm for 30 minutes. The supernatant was discarded, and the sediment was rinsed with deionized distilled water several times and refrigerated. X-ray diffraction and scanning electron microscopy were performed to assess the size of the particles.

###  Preparation of acrylic discs 

 CNP powder at 0.5 w/w%, 1 w/w%, and 2 w/w% concentrations was dissolved in acrylic monomer (Triplex, Ivoclar Vivadent AG, Schaan, Liechtenstein). The acrylic powder and monomer containing different concentrations of CNP were mixed in a 3:1 ratio according to the manufacturer’s instructions. The mixture was poured into a prefabricated mold measuring 11 mm in diameter and 5 mm in thickness. After polymerization, the specimens were allowed to set perfectly for 24 hours at room temperature. Eventually, the samples were removed from the mold and polished using Exsys and Superfine grit discs (3M ESPE, St. Paul, MN, USA). After polishing, the discs measured 10 mm in diameter and 4 mm in thickness. All the samples were gamma-sterilized with a 25-kGy dose.

###  Culture of microorganisms 

 Standard strains of *S. mutans* (ATCC 35668), *S. sanguinis* (ATCC 10556), *L. acidophilus* (ATCC 314), and *C. albicans* (ATCC 14053) were obtained from the Microbiology Department. *S. mutans*, *S. sanguinis*, and *L. acidophilus* cultured in brain heart infusion (BHI) broth (Merck, Germany) were incubated in the presence of 5% CO_2_ (capnophilic conditions using Gas-Pak) while *C. albicans* was incubated in Mueller-Hinton (MH) broth (Merck, Germany) at 37°C for 24 hours.

###  Assessment of antimicrobial properties

 Three tests were performed to assess the antimicrobial effects of CNPs.

*Disc agar diffusion (DAD) test:* For this test, 50 µL of 0.5 McFarland standard concentration of each bacterial suspension containing 1.5 × 10^8^ CFUs/mL of each microorganism was separately prepared using MH broth and streak-cultured on MH agar (Merck, Germany). For DAD testing against *C. albicans*, a concentration of 1.5 × 10^5^ CFUs/mL of *C. albicans* was used. Next, acrylic discs were placed on the plate surface at a 2-cm distance from each other. The plates were then incubated at 37°C for 24 hours under optimal conditions for each microorganism. After incubation, the diameter of growth inhibition zones was measured.

*Eluted component test:*Acrylic discs were immersed in microtubes containing 1 mL of saline solution. After 24 hours, 50 µL of the contents of the microtubes was transferred to another microtube containing 50 µL of the microbial suspension with a final concentration of 1.5 × 10^5^ CFUs/mL for each bacterial species and 1.5 × 10^3^ CFUs/mL for *C. albicans*, separately. Next, the tubes were shaken in an incubator shaker (Eppendorf, Germany) at 200 rpm for 24 hours at 37°C. Eventually, 10 µL of the solution in each microtube was diluted with 90 µL of saline solution. Next, 10 µL of this mixture was cultured in BHI agar (Merck, Germany). Finally, the plates were incubated at 37°C for 24 hours under optimal conditions for each microorganism. The number of colonies was then counted using the “Miles et al method.”^22^ The antimicrobial activity was assessed by the eluted component test at 3 and 7 days using the same method.

*Biofilm inhibition test:* Acrylic discs containing different concentrations of CNPs were immersed in tubes containing microbial suspension with 1.5 × 10^8^ CFUs/mL for each bacterial species and 1.5 × 10^5^ CFUs/mL for *C. albicans* separately and incubated at 37°C for 48 hours under optimal conditions for each microorganism to form biofilm.

 After 48 hours, acrylic discs were removed by sterile forceps and gently shaken and rinsed with saline solution to remove planktonic and loosely attached microorganisms. Acrylic discs were then placed in sterile saline and sonicated at 150 W with a 50-Hz frequency (Bandelin, Germany) for 20 seconds. The number of colonies was then counted using the “Miles et al method.”

 Data were analyzed using SPSS 20 via one-way analysis of variance (ANOVA), repeated-measures ANOVA, and the Bonferroni test. *P*<0.05 was considered the level of significance.

## Results

###  Results of DAD test

 The results of the DAD test showed that incorporating 0.5%, 1%, and 2% concentrations of CNPs did not cause any growth inhibition zone around any microorganism, similar to the control group.

###  Results of eluted component test 

 ANOVA revealed that at all time intervals, all the solutions containing acrylic discs with 0.5%, 1%, or 2% concentrations of CNPs exhibited a significantly lower microbial count for all four microorganisms than the control group.

 At 1 and 7 days, the number of *S. mutans* colonies in the presence of 2% CNPs, was significantly lower than that in the presence of 1% concentration. The same result was achieved with *S. sanguine *on day 1. No other significant difference was noted between 1% and 2% concentrations. At all the three time intervals for all microorganisms, solutions containing 2% CNPs caused significant colony count reductions compared to 0.5% except for *S. sanguinis* on day 3. Conversely, solutions containing 1% CNPs, only resulted in a significant reduction in colony counts of *C. Albicans* with no significant reductions in *S. mutans*, *S. sanguinis*, and* L. acidophilus *colony counts.

Table 1 shows the results of eluted components test for all microbial species at the three time intervals and different concentrations of CNPs. Table 2 presents the percentage of microbial count reductions in liquid media with different concentrations of CNPs.

**Table 1:** Number of microbial colonies (CFUs/mL)×10^5^ at different times in solutions containing acrylic discs

**Day**	**Microbial Species**	**Microbial count mean±SD**			
		**0% CNP**	**0.5% CNP**	**1% CNP**	**2% CNP**
Day 1	*S. mutans*	36.3 ± 4.9^a^	27.6 ± 2.3 ^b^	22 ± 1.7 ^b^	11 ± 2 ^c^
	*S. sanguinis*	41 ± 2.6 ^d^	30.6 ± 4.7 ^e^	23.6 ± 1.1 ^e^	12 ± 2.5 ^f^
	*L. acidophilus*	50.3 ± 2.5 ^g^	32.3 ± 5.1 ^h^	23 ± 3.4 ^hi^	18.3 ± 2.5^i^
	*C. albicans*	0.73 ± 0.2 ^j^	0.49 ± 0.1 ^k^	0.26 ± 0.1 ^l^	0.22 ± 0.1 ^l^
Day 3	*S. mutans*	39.6 ± 3.2 ^a`^	17.6 ± 2 ^b`^	16.6 ± 2 ^b`,c`^	10.3 ± 1.5 ^c`^
	*S. sanguinis*	45.3 ± 4.5 ^d`^	20.6 ± 2.3 ^e`^	17.6 ± 2 ^e`^	15 ± 2 ^e`^
	*L. acidophilus*	72.3 ± 3.7 ^g`^	30 ± 5 ^h`^	22.3 ± 2.5 ^hi`^	16.3 ± 1.1^i`^
	*C. albicans*	0.80 ± 0.1 ^j`^	0.42 ± 0.2^k`^	0.25 ± 0.1 ^l`^	0.18 ± 0.1 ^l`^
Day 7	*S. mutans*	45 ± 3.6 ^a“^	19 ± 2 ^b“^	15 ± 2 ^b“^	6.6 ± 3 ^c“^
	*S. sanguinis*	50.3 ± 5.6 ^d``^	22.3 ± 2 ^e``^	14.6 ± 2 ^e``f``^	11.6 ± 2 ^f``^
	*L. acidophilus*	83 ± 7 ^g“^	26.6 ± 5.8 ^h“^	19.3 ± 0.5^h``i“^	13.3 ± 1.5 ^i“^
	*C. albicans*	0.91 ± 0.4^j“^	0.39 ± 0.2 ^k“^	0.22 ± 0.1^l“^	0.15 ± 0.1^l“^

*Values with similar letters are not significantly different.

**Table 2:** Percentage of reduction in colony counts of the bacteria in solutions containing acrylic discs

**Day**	**Microbial species**	**0.5%**	***P***** value**	**1%**	***P***** value**	**2%**	***P***** value**
		**Percentage of microbial reduction**					
Day 1	*S. mutans*	24%	0.048	39%	0.002	70%	0.000
	*S. sanguinis*	25%	0.019	42%	0.001	69%	0.000
	*L. acidophilus*	36%	0.002	54%	0.000	64%	0.000
	*C. albicans*	33%	0.001	64%	0.000	70%	0.000
Day 3	*S. mutans*	55%	0.000	85%	0.000	74%	0.000
	*S. sanguinis*	54%	0.000	61%	0.000	67%	0.000
	*L. acidophilus*	58%	0.000	68%	0.000	79%	0.000
	*C. albicans*	47%	0.000	68%	0.000	77%	0.000
Day 7	*S. mutans*	58%	0.000	65%	0.000	.001	0.000
	*S. sanguinis*	56%	0.000	71%	0.000	77%	0.000
	*L. acidophilus*	68%	0.000	77%	0.000	84%	0.000
	*C. albicans*	57%	0.000	75%	0.000	83%	0.000

###  Results of biofilm inhibition test 

Table 3 shows biofilm inhibition of different microorganisms by different concentrations of CNPs. According to ANOVA, biofilm inhibition was significantly different for all the microorganisms (*P*<0.001).

**Table 3:** Biofilm inhibition test result (CFUs/mL) by acrylic discs containing different concentrations of CNPs

**Microbial strain**	**Bacterial count (mean±SD)**				***P***** value**
	**0% CNP **	**0.5% CNP **	**1% CNP **	**2% CNP **	
*S. mutans*	426 000 ± 72 000 ^a^	96 000 ± 15 000 ^b^	12 000 ± 3200 ^c^	860 ± 200 ^c^	<0.001
*S. sanguinis*	253 000 ± 87 000 ^d^	113 000 ± 25 000 ^e^	17 000 ± 3600 ^f^	960 ± 150 ^f^	<0.001
*L. acidophilus*	360 000 ± 55 000 ^i^	296 000 ± 41 000 ^i^	29 000 ± 4500 ^j^	2000 ± 200 ^j^	<0.001
*C. albicans*	43 600 ± 1520 ^k^	33 600 ± 6100 ^l^	3100 ± 400 ^m^	220 ± 30 ^m^	<0.001

*Values with similar letters are not significantly different.

 Pairwise comparisons of the different concentrations of CNPs for each microorganism showed that all the concentrations of CNPs led to significant biofilm reduction for all the microorganisms, except the 0.5% concentration of CNPs for *L. *acidophilus. In addition, for all the microorganisms, no significant difference was noted between 1% and 2% concentrations of CNPs. Table 3 shows the detailed pairwise comparison of the groups.

## Discussion

 To the authors’ knowledge, this study was the first to assess the antimicrobial efficacy of acrylic resin containing different concentrations of CNPs. This study evaluated the antimicrobial activity of 0.5%, 1%, and 2% concentrations of CNPs added to acrylic resin against *S. mutans*, *S. sanguinis*, *L. acidophilus*, and *C. albicans*.

 The incorporation of nanoparticles into acrylic resin to induce antimicrobial features has been efficiently performed by the authors and other researchers.^23-25^ We used CNPs in this research based on several studies that have applied antimicrobial and anti-inflammatory properties of curcumin in dentistry. Curcumin mouthrinse has successfully shown anti-gingivitis features comparable to those of chlorhexidine. Furthermore, local injection of curcumin-loaded nanoparticles has effectively inhibited inflammation and bone resorption in periodontal disease. Moreover, curcumin has the potential to attenuate pathogenic traits of* S. mutans* biofilms.^22,26,27^

 Several tests are used to assess the antimicrobial activity of dental materials. The DAD test is commonly used for antimicrobial sensitivity testing. It assesses the ability of antibacterial agents to spread in the agar and cause bacterial growth inhibition. The eluted component test is another method to evaluate antibacterial activity in liquid media. The biofilm inhibition test is also widely used to assess antimicrobial activity against biofilms. It is particularly important in oral medicine studies regarding their major role in the development of dental caries and periodontal disease.^28-30^

 In the present study, the DAD test showed no growth inhibition zone, indicating that CNPs trapped in acrylic could not directly spread into the adjacent areas. Thus, in areas of direct contact of an acrylic plate with the tooth structure, direct diffusion of CNPs would not occur to exert antimicrobial activity. Therefore, these areas require precise oral hygiene observation. The same result was reported in our previous study,^10^ as we assessed the antimicrobial activity of CNPs added to orthodontic composite resins and observed no growth inhibition zone in the DAD test.

 The results of the biofilm inhibition test in our study showed that the microbial counts of all four microorganisms significantly decreased compared with the control group after incorporating different concentrations of CNPs into acrylic resin, except 0.5% for *L. acidophilus*. In the biofilm inhibition test of all the microbial groups, there was a significant difference in the bacterial count of 0.5% compared to the 1% and 2% groups. In addition, increasing the concentration of CNPs from 1% to 2% did not affect biofilm inhibition activity in any microbial group.

 The results of the eluted component test in our study showed that incorporating all concentrations of CNPs into acrylic in a liquid medium such as saliva decreased the microbial counts of all the microorganisms at all time intervals. The eluted component test at 1, 3, and 7 days also revealed an increasing trend for microbial reduction over time. In 7 days, 2% CNPs caused maximum inhibition of *S. mutans*, while there was no significant difference between 1% and 2% CNPs in the bacterial count of *S. sanguinis* during the same period. Considering the equilibrium between *S. sanguinis* and *S. mutans*, an ideal concentration of CNPs should decrease *S. mutans* and increase *S. sanguinis* counts or, at least, not alter the latter.^12,13^ Thus, by increasing the concentration of CNPs from 1% to 2%, the *S. mutans* count decreased while the *S. sanguinis* count remained unchanged in 7 days. Therefore, a 2% concentration of CNPs is more suitable for caries prevention since it significantly reduces *S. mutans* counts and can potentially shift the oral equilibrium to non-cariogenic plaque.

 A comparison of DAD and eluted component tests revealed that CNPs have limited dissemination and spreading in solid media. However, they exert antimicrobial activity in liquid media. Thus, CNPs added to acrylic resin can have optimal antimicrobial activity in patients with a sufficient salivary flow. However, in patients with xerostomia and those taking medications that decrease the salivary flow, the use of artificial saliva is recommended so that they could also benefit from the optimal antimicrobial properties of CNPs added to acrylic resin.

 Further studies on acrylic resins containing CNPs are required to ensure no adverse effect on their mechanical properties. Also, future studies over longer periods are required on other concentrations of CNPs.

## Conclusion

 Incorporating CNPs into acrylic resin was effective against the four types of microorganisms assessed in this study. The increase in the concentration of CNPs from 1% to 2% was in favor of the reduction of *S. mutans* to *S. sanguinis* count ratio, and thus, 2% concentration seems to be suitable for incorporating into the acrylic resin.

## Author Contributions

 PKprepared the acrylic discs, helped with microbiological tests,searched the literature, and contributed to writing the manuscript. MPperformed the microbiological tests and interpreted the data. ASconceptualized the main idea and searched the literature. ABdesigned and supervised the antimicrobial tests. BAprepared CNPs and performed x-ray diffraction and scanning electron microscopy. SAprepared the manuscript, interpreted the data, and searched the literature.

## Funding

 Self-funded.

## Ethics Approval

 This study was approved by the Ethics Committee of Tehran University of Medical Sciences (ethical code: IR.TUMS.DENTISTRY.REC.1396.3679).

## Competing Interests

 The authors do not have any conflict of interest regarding this manuscript.
